# Back to the Future: Historic Insights and Recent Innovations in Pediatric Regional Anesthesia

**DOI:** 10.3390/jcm13226704

**Published:** 2024-11-08

**Authors:** Ashley Mathew, Katrina Kerolus, Nicholas Bitonti, Andrea Guzman, Robert Moore, Sergio Bergese

**Affiliations:** 1 Department of Anesthesiology, Stony Brook University Health Science Center, Stony Brook, NY 11794, USA; 2 Renaissance School of Medicine, Stony Brook University, Stony Brook, NY 11794, USA

**Keywords:** pediatric regional anesthesia, peripheral nerve blocks, adjuvants

## Abstract

Pediatric regional anesthesia is evolving with new peripheral nerve blocks and techniques aimed at improving perioperative pain management. While caudal blocks have long been standard due to their simplicity and low complication rates, newer fascial plane blocks offer comparable efficacy with enhanced nerve coverage tailored to specific surgeries. Moreover, adjuncts like dexmedetomidine and dexamethasone have shown promise in prolonging block duration and enhancing post-operative pain relief and patient satisfaction. The integration of these advancements into clinical practice has yielded significant benefits, including reduced intraoperative fluid requirements, decreased reliance on opioids postoperatively, earlier initiation of enteral nutrition, lower readmission rates, shorter hospital stays, and decreased overall hospital costs. Our review underscores the technical progress and expanding literature supporting the rapid adoption of these impactful regional anesthesia techniques in pediatric care.

## 1. Introduction

Pediatric regional anesthesia is rapidly evolving with the emergence of novel techniques, a greater interest in systematic application of techniques, and an expanding pool of facile providers. This evolving landscape is the product of several long-standing efforts, including investigations of the benefits and safety profile of various techniques. Coupled with new technologies and the development of several novel blocks, these changes have radically altered the landscape and practice of pediatric regional analgesia. This review will provide insight into these novel techniques and the innovative history that produced the current state of practice.

## 2. Background—Establishment of a Risk–Benefit Profile

Regional anesthesia (RA) utilizes the targeted injection of local anesthetics to block sensation in a specific body area, easing pain and limiting the neurohumoral response to stress. Despite potential benefits, adoption requires an understanding of the risk profile. Historically, the wide-spread use of regional anesthesia in pediatrics has been limited by concerns about safety and the availability of skilled providers [1,2,3]. Subsequent advancements in techniques and technology, insight into the possible benefits, and an improved understanding of pediatric anatomy have prompted a growing interest in the use of regional anesthesia.

### 2.1. Benefits

A series of efforts have begun to suggest a variety of benefits of the use of regional anesthesia in pediatric practice. Regional anesthesia offers many clinical and psychological benefits for pediatric patients by reducing postoperative pain and discomfort. Inadequate pain management in newborns and young children can impact their physiologic response following a painful event, potentially leading to both short-term morbidity and promoting long-term neuronal changes that can produce increased pain sensitivity and decreased immune responses [4,5,6]. The analgesia produced by regional techniques may prevent these changes.

The need for general anesthesia may also be decreased or eliminated following the application of a regional technique, contributing to lower hormonal stress responses, positively impacting cardiovascular stability, and lowering perioperative mortality rates [7]. Notably, some evidence suggests that early exposure to general anesthetics may have neurodegenerative properties, such as inducing neuronal apoptosis, potentially contributing to future cognitive or language deficiencies, and making regional techniques a safer alternative in many cases [8,9]. There are ongoing efforts to discern the neurodevelopmental impact of regional techniques [10].

Regional anesthesia also has the ability to minimize the need for opioids [11]. This is of particular significance as early opioid use has been linked to increased risk of chronic use [9]. Reducing opioid use also avoids its associated side effects, such as nausea, vomiting, and constipation, and significantly lowers the risk of respiratory depression.

The application of regional anesthesia may also promote a smoother recovery process, with evidence supporting reduced delirium, shorter hospital stays, earlier participation in physical therapy, and reduced cost of care [12,13,14,15,16].

### 2.2. Risk Profile

Despite the theoretical and established benefits of pediatric regional anesthesia, concerns about safety of these techniques has limited its wide-spread application. This is true historically and in current practice. Two recent surveys have identified significant variability in the acceptance of regional techniques [17,18].

Several large-scale multi-center efforts have allowed for the systematic examination of the safety profile of various regional techniques.

Two major research networks have provided valuable insights into complications in pediatric regional anesthesia. The French Society of Pediatric Anesthesiologists (ADARPEF) conducted two large studies in Europe, one in 1994 and another in 2006, involving 47 hospitals. Meanwhile, the Pediatric Regional Anesthesia Network (PRAN), established in 2007, has gathered data on over 100,000 regional anesthetics from more than 20 hospitals in North America. These networks have been extensively used to investigate and understand the risks and complications associated with regional anesthesia techniques in children.

The overall rate of neurological complications was 2.4 per 10,000 blocks, primarily involving sensory issues that resolved within three months. There were no differences in neurological complications between neuraxial and peripheral nerve blocks, nor among caudal, lumbar, and thoracic epidural blocks. The risk of complications was also similar between single-injection and continuous catheter techniques [19].

Pediatric regional anesthesia is linked to a low incidence of complications [20]. Infection risk can be minimized through strict adherence to aseptic techniques. When coagulation tests show abnormalities, neuraxial and deep peripheral nerve blocks are contraindicated; however, limb peripheral nerve blocks can still be performed safely with ultrasound guidance. Additionally, aspiration prior to the procedure and following simple rules are crucial for reducing complications.

Substantial efforts have been made to validate the safety of well-established techniques, including perineural blocks, epidural analgesia, and specific fascial plane blocks, like the transversus abdominis plane (TAP) block. Additionally, a growing body of literature supports the safety of these methods, especially the pudendal block [21].

## 3. Current Literature

In addition to efforts to establish the risk–benefit profile of specific techniques, ongoing efforts have helped define the impact of regional anesthesia on efforts to systematically improve pediatric perioperative care and pain management.

While research on RA’s benefits in pediatric populations remain limited largely due to challenges in conducting large-scale clinical trials, such as recruitment difficulties, funding constraints, and ethical considerations, there is a growing body of evidence of varying quality.

Several studies have emphasized the pivotal role of integrating regional anesthesia to enhance recovery and promote better outcomes. A qualitative systematic review of randomized control trials has underscored RA’s effectiveness in improving postoperative pain management in pediatric surgical patients [22]. An updated systematic review has reaffirmed these findings, emphasizing RA’s safety and efficacy in improving postoperative analgesia [23]. Specific RA techniques have shown promising results across different surgical procedures, as summarized in Table 1.

Retrospective studies have also highlighted the benefits of RA, including studies demonstrating reduced opioid consumption and improved pain control, with application of RA for pediatric sternotomy patients [35].

A retrospective study was conducted at a pediatric hospital looking at pain scores and opioid exposure in children under 18 years old receiving RA in the context of orthopedic, urologic, or general surgery (Adapted from [36]). Both single-shot and catheter-based regional anesthesia (RA) facilitated opioid-free experiences during surgical procedures and in the post-anesthesia care unit (PACU). However, single-shot RA did not demonstrate any improvement in pain management or reduction in opioid consumption for inpatients after discharge from the PACU. Conversely, catheter-based RA was associated with decreased opioid use during the first 24 h postoperatively; however, beyond this period, there was no significant difference compared to the no-block group, with a notable increase in opioid use observed from 36 to 72 h postoperatively. Overall, RA did not lead to a reduction in cumulative opioid consumption.

A recently published study gathered 69 randomized control trials that compared regional analgesia in pediatric inguinal surgeries (Adapted from [37]). By examining the outcomes of various regional analgesic techniques, it was revealed that the quadratus lumborum (QLB) and transverse abdominis plane (TAP) blocks had the longest time to the first rescue analgesic.

## 4. Current Barriers to Adoption and Application of Pediatric Regional Anesthesia

There is a significant range in the acceptance of regional anesthesia among healthcare providers, with varying levels of comfort influencing their willingness to consider this form of anesthesia [17,18]. Many healthcare facilities need more equipment and institutional support to implement regional anesthesia effectively. Collaborative networks, like the Pediatric Regional Anesthesia Network (PRAN), are helping to address these issues by collecting and presenting data on regional anesthesia from a wide range of participating healthcare centers, with the hope of standardizing techniques and improving both patient outcomes and procedure use [38].

There are notable differences in adoption rates based on the type of healthcare facility and geographical location. Tertiary care centers and specialized pediatric hospitals are more likely to utilize regional techniques than smaller, rural hospitals.

Additionally, the adoption and usage of regional anesthesia in pediatric surgery vary widely across different regions [39]. In high-income countries, its adoption is more prevalent due to better access to resources and skilled providers. Conversely, in low- and middle-income countries, the rate is lower due to various constraints, such as shortages of trained pediatric anesthesia providers and constraints in the supply of the necessary systems and tools. This gap is highlighted by the multi-institutional Anaesthesia Practice in Children Observational Trial (APRICOT) study demonstrating wide variability in the use of RA by country [40].

In light of the growing body of literature suggesting the benefits of the application of RA, regional and international differences in access to pediatric RA may represent a growing source of health care inequality.

## 5. Elimination of Barriers or the “Democratization” of Pediatric Regional Anesthesia

Advancements in technology, education, and novel techniques that can be performed and taught by a larger pool of physicians may promote the wider application of RA and help bridge gaps in the application of RA.

For example, while the integration of ultrasound guidance in regional anesthesia has increased its adoption, it is also increasing the demand for training. Unfortunately, fewer than half of the pediatric anesthesia training programs offer point-of-care ultrasound (POCUS) training [41]. Various programs and initiatives aim to bridge this gap by offering specialized training and certification in pediatric regional anesthesia. For example, some programs use simulated patients and virtual reality programs to provide staff with more practice [42]. Institutions employing these measures have reported increased confidence in performing standard techniques amongst trained staff and higher rates of successful blocks.

## 6. Novel and Emerging Techniques

The ability to apply a group of novel and emerging regional techniques that are rooted in techniques that are familiar to many anesthesiologists will also help bridge resource-driven gaps in access to RA. These techniques include pudendal nerve block, rectus sheath block (RSB), transabdominal plane (TAP) block, quadratus lumborum block (QLB), and erector spinae plane block (ESPB).

### 6.1. Rectus Sheath Block (RSB)

Ultrasound-guided RSB targets the ventral rami of the intercostal nerves supplying the anterior abdominal wall, T9-11. It is typically indicated for hernia surgeries, laparoscopic surgery, or laparotomies. In a prospective study comparing perioperative opioid use in children who received RSB or local anesthetic infiltration of surgical sites found there was a statistical significance between the two groups, with RSB being superior for combatting post-operative pain [43]. In another study assessing the efficiency of RSB in children with umbilical hernias, 22 children were included in the study and received a single injection 0.25 mL/kg of plain 0.25% bupivacaine, and only one child required supplemental morphine for pain [44]. Ultrasound has aided in providing better results because it allows for direct visualization of the target nerve.

### 6.2. Transversus Abdominis Plane (TAP) Block

Ultrasound-guided TAP blocks target the anterior cutaneous branches of segmental thoracolumbar nerves, specifically T7–T9, typically with 1 mL/kg 0.25% bupivacaine for one side or 0.5 mL/kg for bilateral. They have been seen to be useful in upper abdominal surgeries when incision is above the umbilicus. With the guidance of an ultrasound, it is a relatively easy block to achieve adequate pain relief. In a randomized controlled trial, ultrasound guided TAP blocks were assessed for their efficiency in providing post-operative pain relief, minimalizing intraoperative opioid use and overall pain scores. It was found that the total intraoperative fentanyl dose was lower in children who received a TAP block in comparison to the control group. TAP recipients had overall better satisfaction. However both groups had similar durations in hospital stays [45]. In a meta-analysis reviewing TAP blocks and caudal blocks in the pediatric population, TAP blocks was seen to provide increased duration of analgesia close to 1.75 h [46].

### 6.3. Quadratus Lumborum Block (QLB)

Ultrasound-guided QLB targets the nerves located near the quadratus lumborum muscle, which is a posterior abdominal wall muscle. There are several approaches to this block, which include posterior lateral or anterior, and it can cover T7-L2 dermatomes. The typical dosage includes 0.25–0.75 mg/kg of bupivacaine or ropivacaine. This typical block can provide analgesia to the abdominal wall and retroperitoneum and may be utilized for procedures like inguinal hernias, colostomy closure, and orchidopexy. QLB can provide longer and more coverage. In a meta-analysis reviewing the effectiveness of QLB in the pediatric population, three trials showed QLB was more effective in relieving post-operative pain in comparison to caudal blocks. They attributed it to the high absorption of caudal blocks secondary to high vascularity in the epidural space, causing its duration of action to be shortened [47]. In another study, the addition of dexamethasone to bupivacaine in QLB was found to be superior in providing greater analgesia determined by less morphine consumption post-operatively in comparison to IV dexamethasone or bupivacaine alone [48]. Dexamethasone is a corticosteroid that provides anti-inflammatory properties and is believed to inhibit the signal transmission of nociceptive C-fibers.

### 6.4. Erector Spinae Plane Block (ESPB)

Ultrasound-guided ESPB can provide different dermatomal coverage depending on the levels of injection. It can be used to provide coverage of the chest, abdominal, inguinal, and lower limb surgeries depending on the location of injection. Lumbar ESPB is useful in orthopedic hip and proximal femur procedures and lower quadrant abdominal procedures. A study compared ESPB to QLB in children undergoing orchiopexy and inguinal hernia repair. QLB proved to be superior, showing a longer time (27 h vs. 24 h) for the need for rescue analgesia and having less postoperative pain levels in comparison to ESPB [49]. Both groups were seen to provide high satisfaction, with ESPB at 9.7/10 and QLB at 9.5/10 [50].

### 6.5. Pudendal Nerve Block

The pudendal nerve comes from the second, third, and fourth sacral nerve roots. Once it is in the pudendal canal, it first branches off into the inferior rectal (anal) nerves and then splits into the perineal nerve and the dorsal nerve of the penis or clitoris. There are various ways to perform a pudendal nerve block, including different anatomical approaches (transvaginal, transperineal, and perirectal) and techniques (using anatomical landmarks, a nerve stimulator, or ultrasound guidance). Although a pudendal nerve block might take more time and effort to administer, it provides longer-lasting pain relief compared to a caudal block, making it particularly useful for pediatric urologic procedures. A study comparing the effectiveness of pudendal nerve blocks versus caudal blocks for pain management in pediatric hypospadias repair surgery showed that the pudendal block resulted in significantly lower pain scores and a reduced need for additional analgesia within 24 h, with higher family satisfaction compared to the caudal block [27]. Another study compared ultrasound-guided pudendal nerve blocks with dorsal penile nerve blocks for pediatric circumcision. Both techniques provided similar postoperative analgesia, but the pudendal nerve block was associated with higher surgeon satisfaction and increased intraoperative hemodynamic changes and fentanyl use [51].

### 6.6. Ongoing Investigations

Studies currently enrolling continue to explore novel nerve blocks to further optimize postoperative outcomes in pediatric patients. For example, studies are investigating the efficacy of the suprazygomatic maxillary nerve (SZMN) block for tonsillectomy and adenoidectomy, as well as the potential benefits of dexmedetomidine as an adjunct to enhance the analgesic effect of the nerve block [52]. Pediatric patients will be randomized into one of three groups with SZMN block treatment, the SZMN block with a local anesthetic adjunct, and the control cohort with standard of care. Like in previous studies conducted, the postoperative pain scores and opioid consumption will be measured. Additional data measured include the time to discharge, PONV, hospital readmissions, and adverse events related to the nerve block.

Another study aims to look at similar outcomes from the erector spinae block (ESPB) for pediatric scoliosis surgery [53]. The control group will receive standard of care to compare the maximum lidocaine plasma concentrations and inpatient postoperative mobility in addition to the previous studies’ outcome measurements.

## 7. Prolongation of Blocks

The ability to prolong the duration of benefit of RA is a clear goal of practice and an ongoing challenge. Numerous techniques may be beneficial, including the utilization of adjuvants and continuous peripheral nerve catheters. Understanding the impact of these approaches will become increasingly important with greater application and acceptance of RA.

### 7.1. Adjuvants

Local anesthetic adjuncts are defined as the concomitant administration of one or more drugs around the peripheral nerve or plexus [54]. There are numerous advantages to the use of adjuvants. Adding an adjuvant to a local anesthetic can extend its effect, reduce the need for general anesthesia, and prolong the block’s analgesic duration. This can facilitate a smoother emergence from anesthesia, minimize emergence delirium and shivering, and contribute to a more comfortable postoperative period. In ambulatory surgery, it supports early discharge and a pain-free transfer home [55].

The characteristics of the ideal local anesthetic adjunct include availability as a preservative-free solution; chemical compatibility with local anesthetics and accompanying adjuncts; a described mechanism of action; effectiveness in all modalities of peripheral nerve blockade; facilitation of decrease in effective dose of local anesthetic; evidence of dose–response relationship; reduction in the time to onset of motor and sensory blockade; improvement in the quality of sensory blockade; increase in the duration of sensory blockade and no prolongation of motor blockade; differential sensorimotor blockade prolongation; increase in the duration of analgesia; and the absence of systemic adverse consequences, as well as chondrotoxic, myotoxic, and neurotoxic side effects [54].

Important considerations when evaluating an adjuvant are determining whether the adjuvant is available as a preservative-free preparation, whether the overall safety is acceptable, and its toxicity [54]. Common adjuvants used include dexmedetomidine, clonidine, and dexamethasone.

#### 7.1.1. Dexmedetomidine and Clonidine

Clonidine and dexmedetomidine are alpha2-adrenergic agonists. Clonidine is a centrally acting selective partial alpha2-adrenergic agonist, whereas dexmedetomidine is a highly selective alpha2-agonist that is eight times more selective for the α2-receptor than clonidine. However, there is no expression of alpha-2 adrenoceptors on peripheral nerves. Instead, they produce analgesia via three mechanisms:

1. Stimulation of alpha2-adrenergic receptors in the dorsal horn of the spinal cord, thus diminishing pain transmission
2. Increased acetylcholine levels via the release of norepinephrine via the descending noradrenergic pathways, thus causing cholinergic activation in the dorsal horn
3. Intrinsic blockade of Aδ and C fibers

Data regarding the use of dexmedetomidine and clonidine as an adjunct for peripheral nerve blockage in pediatrics are lacking. In 2015, a meta-analysis of five randomized control trials showed that adjuvant use of preservative-free clonidine significantly prolonged block (9.75 h vs. 3.75 h) compared to the use of plain local anesthetic [56]. In 2022, a meta-analysis of eight studies evaluating dexmedetomidine as an adjunct to peripheral nerve blocks in pediatrics showed that it enhanced the analgesic effect of local anesthetics, but the effect was limited to no more than 8 h after surgery [57]. However there was increased risk of bradycardia and increased sedation. In the future, it is crucial to conduct well-designed, standardized, multicenter randomized clinical trials to investigate the potential benefits of alpha 2-adrenergic agonists as supplementary treatments, including identifying the most effective dosage.

#### 7.1.2. Dexamethasone

Dexamethasone, a corticosteroid commonly administered during general anesthesia to prevent postoperative nausea and vomiting (PONV), is also utilized in pediatric anesthesia to reduce edema associated with intubation. The mechanism of action is likely due to the direct inhibition of signal transmission in nociceptive C fibers, local vasoconstriction, and reduced local inflammation [58]. However, its use as a perineural adjunct in children has not been thoroughly investigated.

In 2021, a study of 63 patients, aged 4–14 years, who received supraclavicular, or interscalene block with 2 mL lidocaine 2% and bupivacaine 0.25% (max 2 mg/kg) alone or with either epinephrine or 2 mg dexamethasone showed that patients who received dexamethasone as an adjuvant had a sensory block duration of 9 to 19 h (13.62 ± 3.02), which is, on average, 390 min longer than the group without adjuvant, and 360 min longer than the group with epinephrine as an adjuvant [59].

In a recent study, the administration of intravenous dexamethasone (0.5 g/kg) did not enhance the duration of pudendal nerve block in infants and children aged 6–36 months who underwent hypospadias surgery [60]. Research comparing the effectiveness between perineural and intravenous administration has not definitively established superiority in either adult or pediatric populations.

#### 7.1.3. Other Adjuvants

Adjuvants commonly used for either adults or children (more specifically, neuraxial) include morphine, magnesium, buprenorphine, ketamine, and epinephrine. However, there are limited data available regarding the use of these adjuvants in peripheral nerve blockade settings.

### 7.2. Continuous Peripheral Nerve Blockade

A single-injection peripheral nerve block typically fails to provide consistent pain relief beyond 20 h. Continuous peripheral nerve blockade (CPNB) has emerged as an effective approach to prolong the analgesic effects of a peripheral nerve block for 48 to 72 h following surgery [61]. However, their effectiveness in the pediatric population is not well-established due to limited data. Additionally, logistical challenges include safety in children, minimizing the risks of catheter dislodgement/leakage, patient/parent education, and maintaining appropriate follow-up while the catheter is in place or removed.

Future studies are necessary to study whether the implementation of CPNB has improved outcomes. An observational, multi-institutional study using the Pediatric Regional Anesthesia Network (PRAN) database showed that of 2074 peripheral nerve catheters included in the study, 251 adverse events and complications were recorded, resulting in an overall incidence (95% CI) of complications of 12.1% (10.7–13.5%). The most common complications were catheter malfunction, block failure, infection, and vascular puncture. There were no reports of persistent neurologic problems, serious infection, or local anesthetic systemic toxicity, resulting in an estimated incidence (95% CI) of 0.04% (0.001–0.2%) [62].

Interestingly, an increase in the use of CPNBs within pediatric anesthesia has correlated with a decrease in neuraxial anesthesia [63].

## 8. Summary

Pediatric RA may confer significant physiologic benefits to a vulnerable population. Historically, the wide-spread use of these techniques has been limited by concerns about safety and access to skilled anesthesiologists. Improvements in technology, a greater understanding of the impact of RA, and emerging techniques may allow for more wide-spread application.

Despite the evolving landscape in pediatric RA, there remain significant gaps in the application of the techniques primarily rooted in socioeconomic concerns. Understanding the history, evolution, and current state of pediatric RA, as outlined in this manuscript, could support choices that bridge this gap in access to quality pediatric care. Ultimately, education, research, and technology may help bridge this gap.

Accordingly, there is a critical need for high-quality randomized controlled trials to explore the efficacy of peripheral nerve blocks in children and to compare various adjuncts. Furthermore, research efforts that extend beyond the perioperative period are needed. This is especially true for studies examining techniques that can be readily taught and applied, such as the ESP or pudendal nerve blocks.

## Figures and Tables

**Table 1:** Regional anesthetic techniques and postoperative outcomes.

Regional Anesthesia Technique	Surgical Procedure	Key Findings
Ultrasound-guided rectus sheath block	Appendectomy	Lower pain scores in first 3 h post-surgery; no difference in opioid consumption between groups [24]
	Umbilical hernia	Lower opioid consumption compared to surgical site infiltration; no difference in maximum pain scores [25,26]
Ultrasound-guided pudendal nerve block	Hypospadias	Less postoperative pain and analgesic consumption compared to caudal block; higher patient satisfaction [27,28]
Ultrasound-guided transversus abdominis plane (TAP) block	Inguinal hernia	Decreased opioid consumption at 24 h; lower pain scores compared to control; higher patient satisfaction [29,30,31]
	Lower abdominal procedures	No reduction in 24-h opioid consumption; no difference in postoperative pain scores among different local anesthetic doses [32,33,34]

## Data Availability

No new data were created or analyzed in this study. Data sharing is not applicable to this article.
